# Newborn Screening for G6PD Deficiency in Xiamen, China: Prevalence, Variant Spectrum, and Genotype-Phenotype Correlations

**DOI:** 10.3389/fgene.2021.718503

**Published:** 2021-10-01

**Authors:** Xudong Wang, Zhongmin Xia, Ying He, Xiaoman Zhou, Haixia Zhang, Chunliu Gao, Yunsheng Ge, Xiaofang Cai, Yulin Zhou, Qiwei Guo

**Affiliations:** ^1^ United Diagnostic and Research Center for Clinical Genetics, Women and Children’s Hospital, School of Medicine and School of Public Health, Xiamen University, Xiamen, China; ^2^ Xiamen Newborn Screening Center, Women and Children’s Hospital, School of Medicine, Xiamen University, Xiamen, China; ^3^ School of Medicine, Xiamen University, Xiamen, China; ^4^ School of Public Health, Xiamen University, Xiamen, China

**Keywords:** G6PD deficiency, newborn screening, variant spectrum, genotype-phenotype correlation, cold-chain transportation

## Abstract

**Background:** Glucose-6-phosphate dehydrogenase (G6PD) deficiency is a common inherited enzymatic defect. The purpose of this study was to evaluate the profile of G6PD deficiency and investigate the factors associated with the accuracy of newborn screening (NBS) in Xiamen, China.

**Methods:** A total of 99,546 newborns were screened by modified fluorescent spot test at the Women and Children’s Hospital, Xiamen University. High-risk neonates were recalled for diagnosis by either a measurement of G6PD activity or genetic testing for the presence of pathogenic *G6PD* variants using a quantitative G6PD enzymatic assay or the MeltPro® G6PD assay, respectively.

**Results:** In the first-tier screening, 1,256 newborns were categorized as high risk. Of these, 1,051 were diagnosed with G6PD deficiency, indicating a prevalence of 1.39% in Xiamen, China. Among the 1,013 neonates who underwent genotyping, 851 carried hemizygous, heterozygous, homozygous, or compound heterozygous variants, for a positive predictive value (PPV) of 84.01%. In total, 12 variants and 32 genotypes were identified, and the six most common variants were c.1376G>T, c.1388G>A, c.95A>G, c.1024C>T, c.871G>A, and c.392G>T, which accounted for approximately 94% of the identified alleles. Different variants showed characteristic enzymatic activities, although high phenotypic heterogeneity was observed for each variant. The use of cold-chain transportation significantly improved the PPV of NBS.

**Conclusions:** We determined the profile of G6PD deficiency in Xiamen, including the prevalence, variant spectrum, and genotype-phenotype correlations and confirmed that maintaining a low temperature during sample transport is essential to ensure the high screening accuracy of NBS. Our data provides epidemiological, genotypic, phenotypic, and clinical practice references to standardize future interventions for G6PD deficiency.

## Introduction

Glucose-6-Phosphate Dehydrogenase (G6PD) deficiency (OMIM #300908), which was first described by Carson (1970), is an X-linked, incomplete-dominant, genetic disease, with an estimated global prevalence of 4.9% that affects more than 500 million individuals worldwide (Nkhoma et al., 2009; Luzzatto et al., 2020). It is more prevalent in some regions, including Africa, South Europe, the Middle East, Southeast Asia, and the Mediterranean. Clinically, the most common presentations of G6PD deficiency are Neonatal Jaundice and Acute Hemolytic Anemia, which can be triggered by ingestion of fava beans, exposure to certain oxidative drugs, or infection (Cappellini and Fiorelli 2008; Peng et al., 2015). Based on residual enzyme activity, the World Health Organization (WHO) has categorized G6PD variants into five classes (class I to V) (WHO 1989).

The pathogenic gene of G6PD deficiency is *G6PD*, which is located on Xq28 and consists of 13 exons and 12 introns. To date, more than 200 pathogenic *G6PD* variants have been identified, most of which are single nucleotide substitutions. The variant spectrum differs among regions and populations (Minucci et al., 2012; Peng et al., 2015; Gómez-Manzo et al., 2016a; Chen et al., 2018; He et al., 2020). Pathogenic *G6PD* variants disrupt enzyme synthesis, stability, or function, resulting in an enzyme deficiency in the red blood cells and the related clinical presentations (Gómez-Manzo et al., 2014; Gómez-Manzo et al., 2016b; Ramírez-Nava and Ortega-Cuellar, 2017; Hwang and Mruk 2018; Bahr et al., 2020). Among the 200 + pathogenic variants, at least 35 variants have been reported in the Chinese population (Liu et al., 2020). Variants c.1376G>T, c.1388G>A, c.95A>G, c.1024C>T, and c.871G>A account for approximately 95% of G6PD deficiency cases in the Chinese population, whereas variants c.392G>T, c.487G>A, c.517T>C, c.592C>T, and c.1004C>A account for approximately 3.5% of the cases (Liu et al., 2020).

Like most genetic disorders, G6PD deficiency cannot be cured. However, Newborn Screening (NBS) is a simple and cost-effective strategy to identify neonates at high risk of this disease, which facilitates early diagnosis and timely intervention. Previous studies have demonstrated that G6PD deficiency is prevalent in parts of South China, such as Guangdong, Guangxi, Yunnan, and Hainan. Accordingly, NBS for G6PD deficiency was initiated in South China, and is currently implemented in an extensive region of China due to its clinical importance for the diagnosis of neonatal jaundice and hemolysis (Moiz et al., 2012; Howson et al., 2018). Xiamen, a city in Fujian province in Southeast China, with a total area of 1,700.61 km^2^ and a population of approximately 5.16 million, established its NBS center in 2002 for the screening, recall, confirmatory diagnosis, treatment, and follow-up of inherited metabolic diseases. NBS for G6PD deficiency was initiated in Xiamen in 2007, and citywide screening was implemented in April 2017. To date, more than 300,000 neonates have been screened in Xiamen.

In this study, we reported the results of NBS and subsequent diagnosis for G6PD deficiency in a total of 99,546 neonates at Xiamen NBS center between January 2017 and May 2018. We determined the profile of G6PD deficiency in Xiamen city, including the prevalence, *G6PD* variant spectrum, and genotype-phenotype correlations, and also revealed the effect of temperature during sample transport on screening accuracy.

## Materials and Methods

### Flowchart for Newborn Screening and Diagnosis of Glucose-6-Phosphate Dehydrogenase Deficiency

**FIGURE 1:**
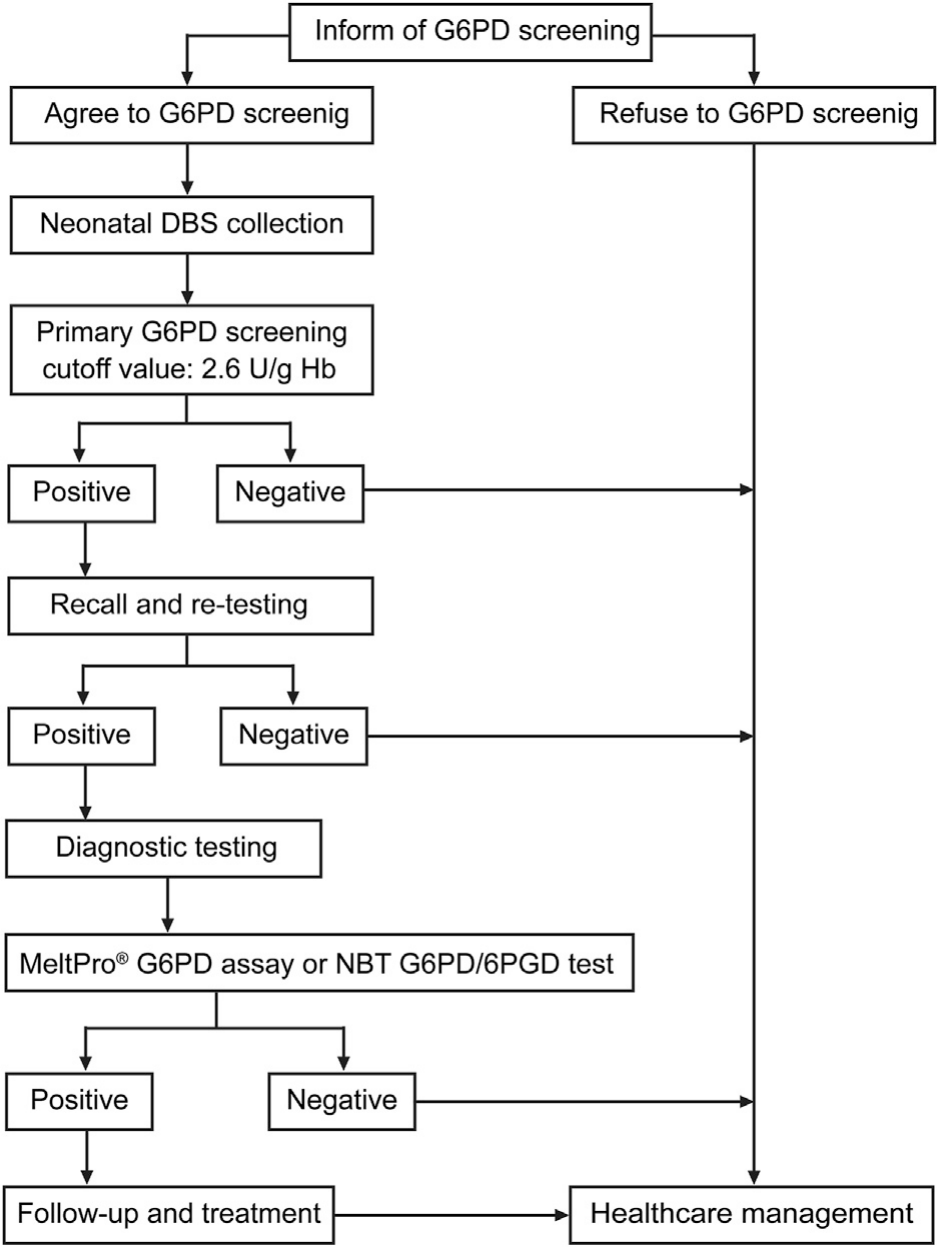
Flowchart for screening newborns and diagnosis of G6PD deficiency. Recall and re-testing were conducted using a new dried blood spot (DBS), whereas confirmatory testing was conducted using a peripheral blood sample from the patients.

In this descriptive, retrospective study, 99,546 newborns (54,101 males and 45,445 females) were screened for G6PD deficiency during the period from January 2017 to May 2018 at the Xiamen NBS center in the Women and Children’s Hospital. Neonatal heel prick blood samples were collected from each newborn between the third and seventh days of life and spotted on Whatman 903 filter paper (Guthrie card). After drying at room temperature, the Dried Blood Spot (DBS) samples were packed and delivered to the Xiamen NBS center the next day. Before August 2017, the DBS samples from other hospitals were transported *via* Express Mail Service (EMS). Beginning in August 2017, EMS transportation was replaced with cold-chain transportation. The flowchart for G6PD deficiency diagnosis is shown in Figure 1. First, DBS samples were collected for screening. Then, the subjects with positive screening results, who were considered at high risk for G6PD deficiency, were recalled, and subjected to diagnostic testing. For diagnosis, 1 ml whole blood sample was collected. The research protocol was approved by the Ethics Committee of Women and Children’s Hospital, School of Medicine, Xiamen University. Written informed consent was obtained from all parents or guardians.

### Newborn Screening Test for Glucose-6-Phosphate Dehydrogenase Activity

In China, NBS for G6PD activity consists of a quantitative evaluation of Nicotinamide Adenine Dinucleotide Phosphate (NADPH) levels, which is a substrate of G6PD. In our study, a modified Fluorescent Spot Test (FST) was used to assess G6PD activity in DBS samples using the Neonatal G6PD screening kit (Perkin Elmer, Waltham, Massachusetts, United States) according to the manufacturer’s protocol. Briefly, G6PD calibrators, G6PD controls, and DBS samples were simultaneously incubated with 100 µl of dissolved G6PD substrate for 30 min at room temperature with gentle mixing. Then, 200 µl of copper reagent was added to each well. After gentle mixing, 200 µl of the mixture was quickly transferred to a new white microplate. Within 15 min of adding the copper reagent, the fluorescence in each well was measured with a DEFIA-1420 semi-auto time-resolved fluoroimmunoassay analyzer (Wallac Oy, Turku, Finland). Based on the manufacturer’s recommendation, the cutoff value of G6PD activity for G6PD deficiency was set at 2.6 U/g Hb. Neonates with G6PD activity <2.6 U/g Hb were re-tested using another DBS. As shown in Figure 1, when the G6PD activity was <2.6 U/g Hb in the repetitive test, the newborn was recalled for diagnostic testing.

### Diagnostic Testing for Glucose-6-Phosphate Dehydrogenase Deficiency

Newborns with positive results in the screening test were recalled for diagnostic testing of G6PD activity or *G6PD* variants. Due to lack of guidelines or professional consensus at the time of the study, after comprehensive genetic counseling, each guardian was allowed to choose between G6PD activity measurement and *G6PD* genotyping for the diagnosis. G6PD activity was evaluated by determining the ratio of G6PD to 6-Phosphogluconate Dehydrogenase (6PGD) using the Nitroblue Tetrazolium (NBT) G6PD/6PGD test (KingMed Diagnostics, Fuzhou, China) according to the manufacturer’s protocol. A patient with a G6PD/6PGD ratio <1.0 was considered to be G6PD deficient. For *G6PD* Genotyping, Genomic DNA was extracted from 200 µl of whole blood with the Super/HF16 plus DNA Extraction System (MagCore, Xiamen, China). After quantification with a Nanodrop 2000^TM^ Spectrophotometer (Thermo Fisher Scientific, Wilmington, DE, United States), 10 ng of DNA was analyzed using the MeltPro® G6PD Genotyping Kit (Zeesan Biotech, Xiamen, Fujian, China) to detect 12 common *G6PD* variants: c.95A>G, c.383T>C, c.392G>T, c.487G>A, c.517T>C, c.592C>T, c.871G>A, c.1004C>A, c.1024C>T, c.1360C>T, c.1376G>T, and c.1388G>A. The reaction was performed on a SLAN-96 real-time PCR system (Hongshi, Shanghai, China). The PCR amplification and melting curve conditions are described in detail in Supplementary Table S1. Melting curves were obtained by plotting the negative derivative of fluorescence to temperature versus temperature (*-dF*/*dT*), and the *T*_*m*_ values were automatically obtained from the melting curves using SLAN 8.0 software (Hongshi).

### Statistical Analysis

Statistical analyses were performed using the SPSS 20.0 Statistical Software (SPSS Inc., Chicago, IL, United States). *t*-test and chi-square test were used to evaluate the effect of transport temperature on screening accuracy. The Mann–Whitney *U*-test and Kruskal–Wallis test were used for parametric and non-parametric comparisons. A *p* value less than 0.05 was considered statistically significant.

## Results

### Overview of Newborn Screening and Diagnosis of Glucose-6-Phosphate Dehydrogenase Deficiency

**FIGURE 2:**
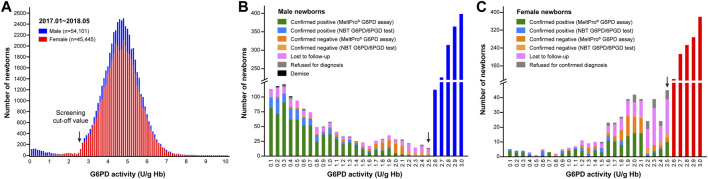
G6PD activity results from newborn screening. **(A)** Enzyme activities in male (blue bars) and female (red bars) newborns. The distribution of G6PD activities <2.6 U/g Hb differed between males **(B)** and females **(C)**. The black arrows indicate the cutoff value (2.6 U/g Hb).

From January 2017 to May 2018, a total of 118,123 live neonates were born in Xiamen (Supplementary Figure S1A). Of these, 99,546 (54,101 males and 45,445 females) were screened for G6PD deficiency (Supplementary Figure S1B). The coverage for NBS of G6PD deficiency in Xiamen was approximately 84.27% (99,546/118,123; Supplementary Figure S1C). As shown in Figure 2, for NBS, the mean, median, and standard deviation for G6PD activity were 4.6, 4.7, and ± 1.0 U/g Hb for male newborns, and 4.7, 4.7, and ± 0.9 U/g Hb for female newborns, respectively. Using a cutoff value of 2.6 U/g Hb for the first screening, 1,653 newborns (1,243 males and 410 females) were considered to be at high-risk for G6PD deficiency, yielding screen positive rates of 1.66% (1,653/99,546), 2.30% (1,243/54,101), and 0.90% (410/45,445) for all, male, and female newborns, respectively.

**FIGURE 3:**
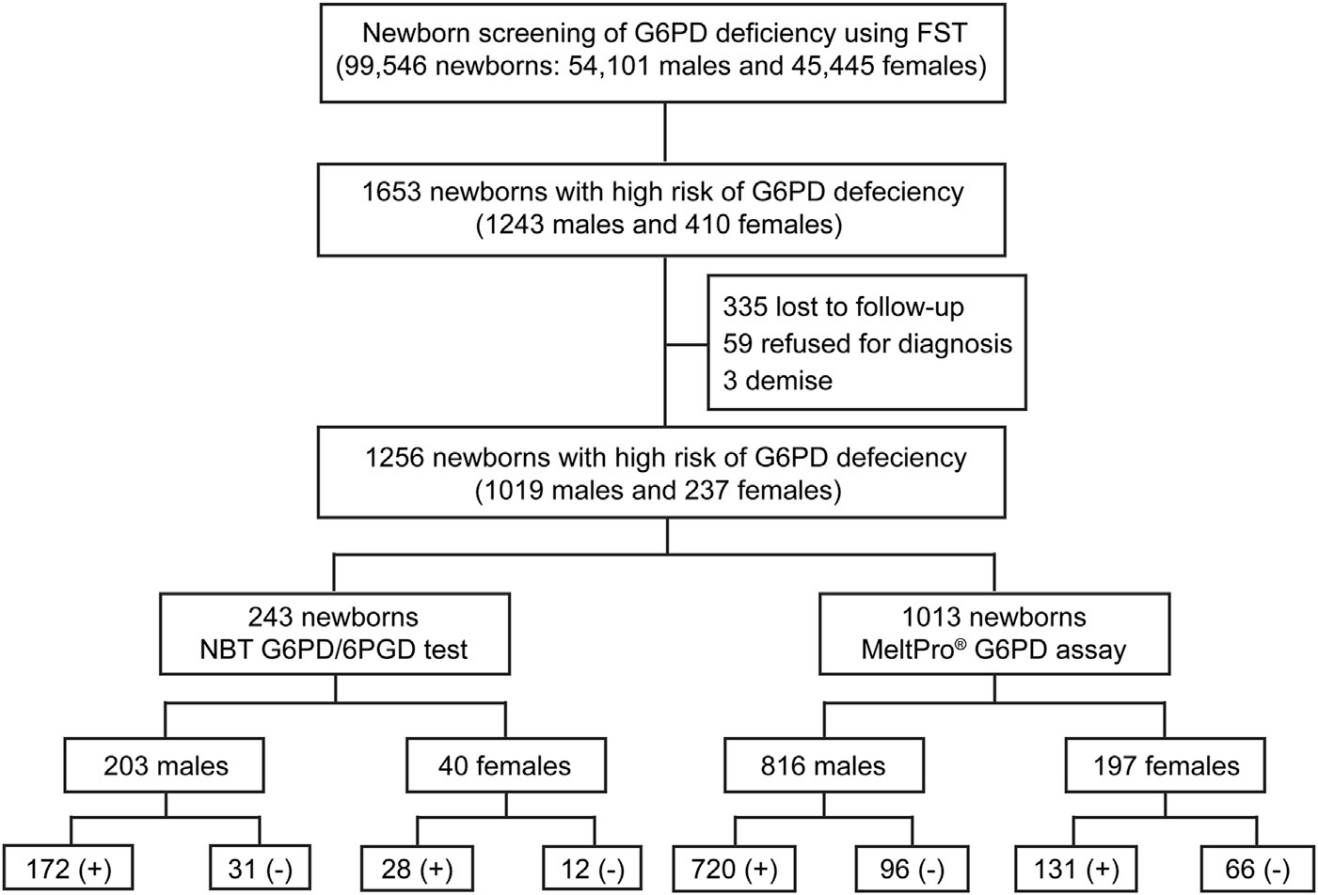
Flowchart showing an overview of the results from newborn screening and diagnostic tests for G6PD deficiency. FST, fluorescent spot test; G6PD/6PGD (+), G6PD deficiency positive; G6PD/6PGD (−), G6PD deficiency negative; MeltPro^®^ G6PD assay (+), target variants detected; MeltPro^®^ G6PD assay (−), no target variants identified.

As shown in Figure 3, of the 1,653 high-risk newborns, 1,256 (1,019 males and 237 females) were successfully recalled for subsequent diagnosis using either the NBT G6PD/6PGD test or *G6PD* genotyping, for an overall recall rate of 75.98% (1,256/1,653) (Figure 2). The remaining 394 newborns were either lost to follow-up or parental refusal of diagnosis (Figures 2B,C). Unfortunately, three male neonates died before diagnosis; their G6PD activities were 0.2, 0.3, and 0.4 U/g Hb (Figure 2B). In the subsequent diagnosis, 243 newborns were tested using the NBT G6PD/6PGD assay, and 200 (172 males and 28 females) of them were diagnosed with G6PD deficiency (Figure 3), for a Positive Predictive Value (PPV) of 82.30% (200/243). The remaining 1,013 newborns were tested using the MeltPro® G6PD genotyping assay, and 851 (720 males and 131 females) of them carried hemizygous, heterozygous, homozygous, or compound heterozygous *G6PD* variants (Table 1 and Figure 3), indicating a PPV of 84.01% (851/1,013). The most prevalent variants were c.1376G>T (45.0%) and c.1388G>A (22.8%), followed by c.95A>G (9.0%), c.1024C>T (7.5%), c.871G>A (5.0%), c.392G>T (4.7%), c.1360C>T (2.9%), c.487G>A (2.3%), c.517T>C (0.3%), c.383T>C (0.2%), c.592C>T (0.1%), and c.1004C>A (0.1%) (Table 1). Ultimately, 892 male and 159 female newborns were diagnosed with G6PD deficiency. Based on these data, the prevalence of G6PD deficiency in Xiamen was estimated to be 1.39% (the number of total neonates multiplied by the recall rate and divided by the number of diagnosed patients). There was no significant difference in the PPV of NBS as determined by diagnosis using the G6PD activity test or *G6PD* genotyping (χ^2^ = 0.416, *p* = 0.519), and the overall PPV for NBS of G6PD deficiency was 83.68% (1,051/1,256).

**TABLE 1:** Allele frequencies of *G6PD* variants in 851 neonates.

Name	Nucleotide change	Protein change	Male hemizygote	Female			Sum of alleles	Frequency (%)
				Heterozygote	Homozygote	Compound heterozygote^a^		
Canton	c.1376G>T	p.R459L	311	59	10	4	394	45
Kaiping	c.1388G>A	p.R463H	170	20	3	4	200	22.8
Gaohe	c.95A>G	p.H32R	68	10	—	1	79	9
Chinese-5	c.1024C>T	p.L342F	59	3	2	—	66	7.5
Viangchan	c.871G>A	p.V291M	35	2	2	3	44	5
Quing Yan	c.392G>T	p.G131V	37	2	1	—	41	4.7
Union	c.1360C>T	p.R454C	17	6	1	—	25	2.9
Mahidol	c.487G>A	p.G163S	18	2	—	—	20	2.3
Nankang	c.517T>C	p.F173L	2	1	—	—	3	0.3
Vanua lava	c.383T>C	p.L128P	1	1	—	—	2	0.2
Coimbra	c.592C>T	p.R198C	1	—	—	—	1	0.1
Fushan	c.1004C>A	p.A335D	1	—	—	—	1	0.1
Total	—	—	720	106	19	12	876	100

a The genotypes of compound heterozygotes were c.1376G>T/c.1388G>A (2 patients), c.871G>A/c.1388G>A (2 patients), c.871G>A/c.1376G>T (1 patient), and c.95A>G/c.1376G>T (1 patient), respectively.

### Genotype-Phenotype Correlation of Glucose-6-Phosphate Dehydrogenase Variants

**FIGURE 4:**
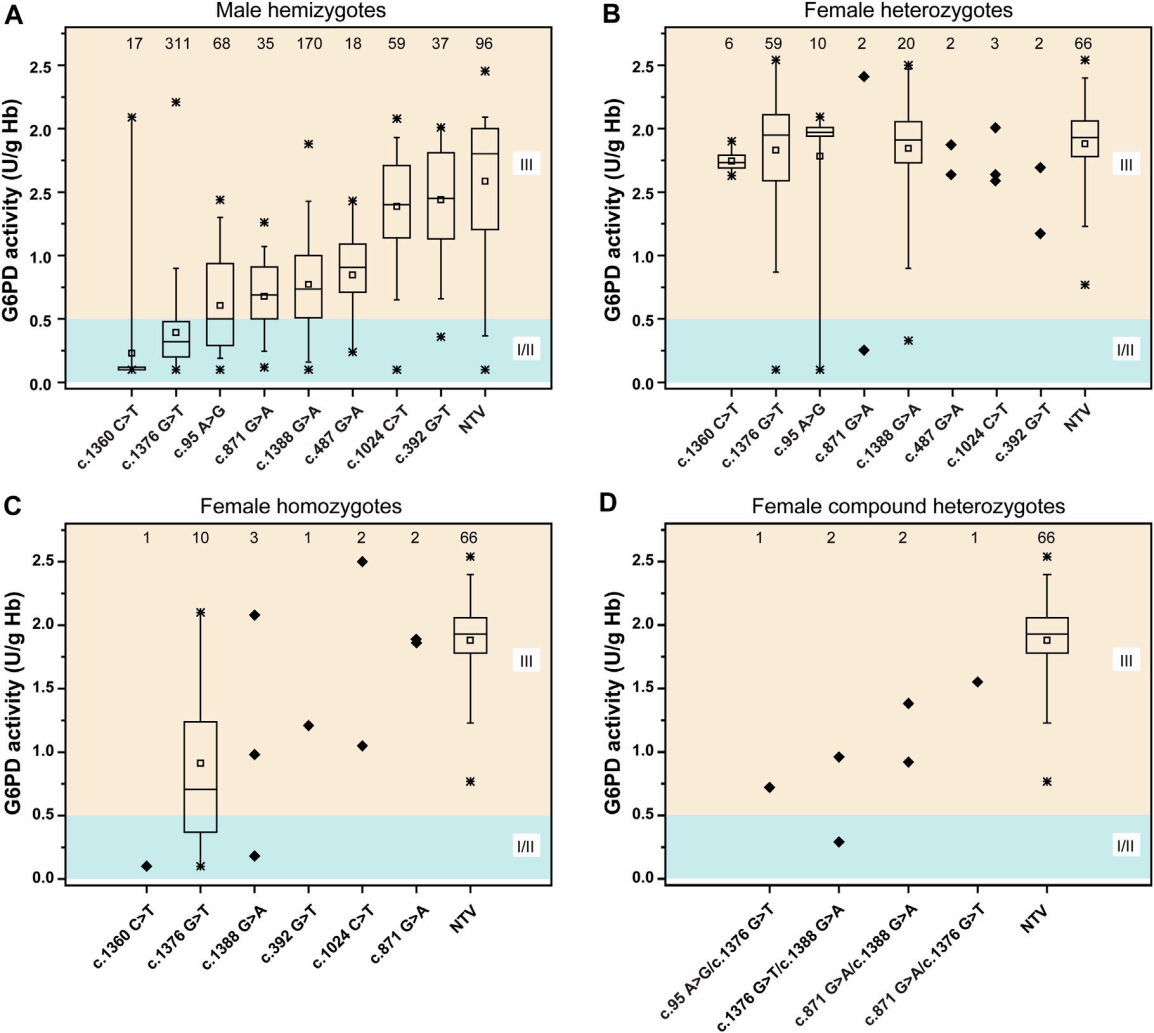
Distribution of the G6PD activities of different variants. **(A)** G6PD activities for male hemizygotes. **(B)** G6PD activities for female heterozygotes. **(C)** G6PD activities for female homozygotes. **(D)** G6PD activities for female compound heterozygotes. According to WHO standard, the residual enzyme activity ≤0.5 U/g Hb (10% of normal) was grouped into class II (severe deficiency); the residual enzyme activity between 0.5–2.8 U/g Hb (10–60% of normal) was grouped into class III (moderate deficiency), the residual enzyme activity between 2.8–7.1 U/g Hb (60–150% of normal) was grouped into class IV (normal). Class I, which includes severely deficient variants associated with chronic nonspherocytic hemolytic anemia (CNSHA). Statistical analysis: the line within the box denotes the median, the square within the box denotes the mean, the horizontal borders of each box denote the 25th and 75th percentiles, the whiskers denote the 5th and 95th percentiles, and the stars denote the maximum and minimum. The samples were denoted as diagonal squares when statistical analysis cannot be performed due to the small sample size. NTV, negative for target variants.

A total of 1,013 newborns were genotyped using the MeltPro® G6PD assay. The distribution of *G6PD* genotypes and their corresponding G6PD activities are shown in Figure 4 and Supplementary Table S2. G6PD activities in hemizygous males were significantly lower than those in genetically negative males (*p* < 0.0001, Supplementary Figure S2A). Differences in G6PD activity were observed among the different variants, and the lowest enzymatic activity was observed among neonates carrying the c.1360C>T variant, followed by c.1376G>T, c.95A>G, c.871G>A, c.1388G>A, c.487G>A, c.1024C>T, and c.392G>T (Figure 4A). In contrast, no significant difference in G6PD activity was observed between heterozygous females and genetically negative females (*p* = 0.487, Supplementary Figure S2B). In terms of specific variants, G6PD activity levels in c.1376G>T carriers were lower in hemizygous males and homozygous females than in Heterozygous females (Supplementary Figure S2C).

**FIGURE 5:**
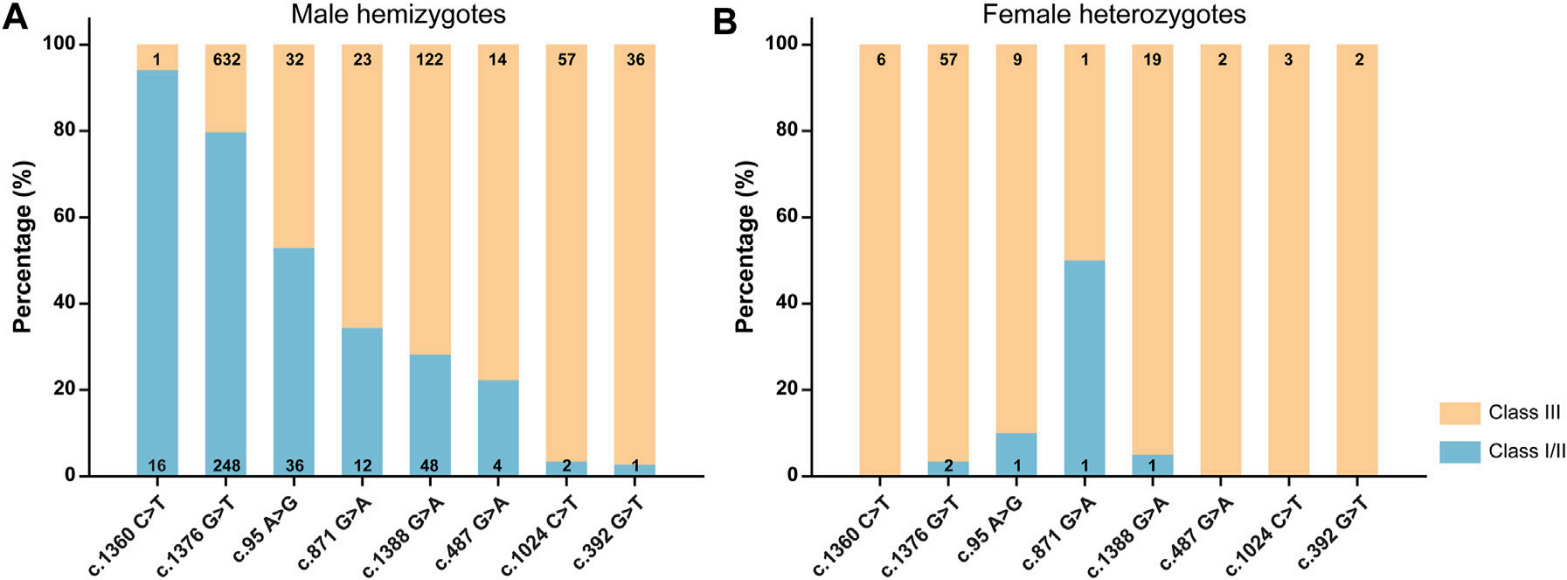
Classification of *G6PD* variants based on the World Health Organization criteria for male hemizygotes **(A)**, female heterozygotes **(B)**. The numbers in the histograms represent the number of newborns with each variant.

We categorized the variants based on WHO classification criteria (Figure 5 and Supplementary Table S3). The results showed that most variants were categorized into different classes, suggesting high phenotypic heterogeneity for *G6PD* variants.

### Effect of Temperature on the Screening Accuracy

**FIGURE 6:**
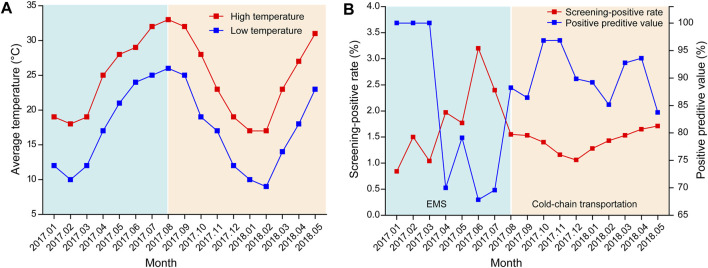
Effect of temperature on the screening accuracy **(A)** Monthly average temperature in Xiamen from January 2017 to May 2018 (data were obtained from https://www.tianqi.com/xiamen/). **(B)** Screening-positive rates and positive predictive values of newborn screening from January 2017 to May 2018. EMS, express mail service.

Before August 2017, the DBS samples from other hospitals were transported *via* EMS; however, from August 2017, the samples were delivered *via* cold-chain transportation. As shown in Figure 6, before August 2017, with the increase in temperature, the positive rate of NBS increased while its PPV decreased. In contrast, a more stable positive rate and PPV, which were independent of the outside temperature, were observed from August 2017. Statistical analysis demonstrated that while there was no significant difference in the daily average temperature between July and August 2017 (*p* = 0.098), the PPV of NBS was significant higher in August than in July (χ^2^ = 9.770, *p* = 0.002).

## Discussion

G6PD deficiency is an inherited metabolic disease that is prevalent in South China, especially in the Southern Regions of the Yangtze River (Yang et al., 2015). In a recent nationwide investigation, the prevalence of G6PD deficiency was 0.67–0.77% in China, ranging from 0.008 to 3.387% in each studied province (Liu et al., 2020). In our study, the prevalence of G6PD deficiency in Xiamen was estimated to be 1.39%, which is higher than the prevalence in most provinces, even higher than the combined prevalence in South China (0.95%), highlighting the importance of NBS for G6PD deficiency in this city.

One of the factors affecting the heterogeneous prevalence of *G6PD* variants in China is the difference in the genetic background, i.e., regional and ethnic differences (Liu et al., 2020). Therefore, genotyping of *G6PD* is essential not only for diagnosis but also for epidemiological investigations and genotype-phenotype correlation studies. In our study, the MeltPro® G6PD assay, which detects 12 common *G6PD* variants in China, was used for *G6PD* genotyping (Xia et al., 2016). Compared with other molecular methods (Tian et al., 2017; Lin et al., 2018; Chiu et al., 2019), the MeltPro® G6PD assay is a rapid, high-throughput, easy-to-use, and cost-effective assay with a high clinical sensitivity and specificity (Xia et al., 2016). The spectrum of *G6PD* variants in Xiamen was similar to that previously reported in studies performed in the same province (Fujian province) (Chen et al., 2018), suggesting regional consistency in genetic background. Specifically, 12 variants and 32 genotypes were identified, and the six most common variants, c.1376G>T, c.1388G>A, c.95A>G, c.1024C>T, c.871G>A, and c.392G>T, accounted for approximately 94.0% of the identified pathogenic alleles in Xiamen. Before the first consensus was published in March 2019 (Consensus on laboratory testing for newborn screening and diagnosis of glucose-6-phosphate dehydrogenase deficiency, reference in Chinese), there were no guidelines regarding the use of diagnostic methods for G6PD deficiency; thus, clinical diagnosis was based on either enzymatic activity or genotyping. Theoretically, targeted variant-based genotyping would miss some variants that were outside the targets, leading to false negative results. However, according to the literature, the MeltPro® G6PD assay diagnosed 97.7% of cases with reduced G6PD activity (Xia et al., 2016) and 96.1% of NBS high-risk cases (Liu et al., 2020), suggesting that the false negative rate would be very low. Moreover, the PPV of NBS calculated based on the results of the G6PD activity measurements and genotyping did not differ significantly in our study, indicating the consistency of these two diagnostic methods. However, more comprehensive genotyping would be desirable in future studies or in clinical practice, particularly in the era of high-throughput sequencing where hundreds or even thousands of genes could be screened simultaneously at a reasonable cost (Luo et al., 2020). Simultaneous evaluation of G6PD activity and genotype will advance our knowledge of the genotype-phenotype correlations in G6PD deficiency.

Investigating genotype-phenotype correlations is of great significance for genetic counseling, prognosis prediction, and management of G6PD deficiency (Jiang et al., 2012; Powers et al., 2018). Generally, in this study, each *G6PD* variant showed a characteristic enzymatic activity (Figure 4A). G6PD activities were significantly lower in genetically positive males (with a known pathogenic variant) than genetically negative males, indicating that most genetically negative males may not have G6PD deficiency, further suggesting the high clinical sensitivity of genetic diagnosis. In contrast, no significant difference in G6PD activity was observed between heterozygous females and genetically negative females (*p* = 0.386) (Figure 4B), which could be attributed to random X-inactivation in females and the compensating effect of the unaffected allele (Zaffanello et al., 2004). Similarly, as expected for an X-linked condition, G6PD activities were lower in hemizygous males and homozygous females than in heterozygous females. A report from the WHO working group categorized *G6PD* variants into five classes based on enzymatic activity and the related clinical manifestations (WHO 1989). Specifically, class I variants are associated with the severe manifestation, Chronic Non-Spherocytic Hemolytic Anemia (CNSHA); class II variants, which include the common mediterranean and severe oriental variants, have less than 10% residual enzyme activity but are not associated with CNSHA. Class III variants, which include the common African variants, show 10–60% residual enzymatic activity. Class IV and V variants are associated with normal and increased enzyme activity, respectively. However, although characteristic enzymatic activities have been suggested for different *G6PD* variants (Figure 4A), high phenotypic heterogeneity is observed for a specific variant, not only among male hemizygotes, female homozygotes, and female heterozygotes but also among male hemizygotes themselves (Figure 5). For example, c.1388G>A and c.871G>A were categorized as class II variants based on the WHO classification (Beutler 1991). However, based on the same principle, 71.8% cases with c.1388G>A and 65.7% cases with c.871G>A in our study displayed residual enzymatic activities associated with class III variants (Figure 5). Whereas, in a study conducted in Shenzhen city, a city in South China that is just 600 km from Xiamen, more than 95% of cases with these two variants displayed residual enzymatic activities associated with class II variants (He et al., 2020). Many factors could be contributive to the highly heterogeneous phenotypes among individuals with the same genotype; however, the exact mechanism remains to be elucidated. For example, the number and the physiological status of red cells could be different among individuals, which could cause the variation of the G6PD activities tested with the DBS samples (Luzzatto et al., 2020). In addition to red cells, other components in blood could also influence the test results. Moreover, the transcriptional level of impaired G6PD could be different among individuals, thus result in various G6PD activities (Makarona et al., 2014). In this sense, the clinical significance of *G6PD* variation classification is limited and it should be reasonable to revise the WHO classification to one in which genotypes are removed, while phenotypes, such as enzymatic activity and disease severity, are retained, which are commonly used to classify other monogenic disorders, such as spinal muscular atrophy and androgen insensitivity syndrome (Hughes et al., 2012; Kolb and Kissel 2015).

Notably, the overall PPV of NBS in our study was lower than that in other studies, which led us to investigate the factors that affect screening accuracy. Eventually, environmental temperature was confirmed to play an important role in screening accuracy (Figure 6). According to previous studies (Flores et al., 2017), G6PD activity, as determined using DBS, decreases with increasing temperature, humidity, and storage time. Moreover, samples stored at 4°C for up to 1 month or at −20°C for 1 year retained more than 90% enzymatic activity. As G6PD deficiency is highly prevalent in tropical and sub-tropical regions, which have relatively high temperature and humidity, quality controls for sample collection, transportation, and storage are essential to maintain screening accuracy. As shown in our study, the use of cold-chain transportation, which stabilized enzymatic activity by providing an environment with a consistently low temperature and humidity, significantly improved the PPV of NBS (Figure 6). Unfortunately, as humidity data were lacking in our study, we were unable to evaluate its effect on the screening accuracy. However, the DBS samples in our study were stored in zip-lock bags and transferred in a closed container, and thus should have been less affected by high humidity.

To further improve the efficiency of screening, diagnosis, and treatment of neonatal inherited metabolic diseases in our city, the following strategies should be used in future NBS programs: First, the cut-off values should be revised based on a larger cohort of neonates to improve screening accuracy while reducing unnecessary recalls and the financial and psychological burdens for patients. Second, closed-loop management of NBS programs should be implemented and the duration from sample collection, through delivery, receipt, and detection, to the issuing of reports should be shortened, which will facilitate more timely diagnosis and treatment while minimizing neonatal morbidity and mortality. Third, government supports for the screening, diagnosis, and treatment of neonatal inherited metabolic disease should be strengthened. Since July 1, 2020, NBS for phenylketonuria, congenital hypothyroidism, G6PD deficiency, and congenital adrenal hyperplasia has been included in the Xiamen Government Benefit Project, which will effectively promote NBS programs in this region. Finally, health education regarding the significance of NBS should be advanced to minimize both the number of cases lost-to-follow-up and diagnosis refusal.

In conclusion, we systematically analyzed the results of NBS and diagnosis of G6PD deficiency in a large cohort of neonates in Xiamen. The profile of G6PD deficiency, including the prevalence, variant spectrum, and genotype-phenotype correlations, in Xiamen was described, which highlights the importance of NBS, genetic counseling, accurate classification, and intervention for this disease. Moreover, we showed that maintaining a stable low temperature and low humidity during sample transport is essential to ensure high screening accuracy for G6PD deficiency. Therefore, our data provide epidemiological, genotypic, phenotypic, and clinical practice references for the standardization of future interventions for G6PD deficiency.

## Data Availability

The original contributions presented in the study are included in the article/Supplementary Material, further inquiries can be directed to the corresponding authors.
