# Supplementary material for: Newborn Screening for G6PD Deficiency in Xiamen, China: Prevalence, Variant Spectrum, and Genotype-Phenotype Correlations
Source: Front Genet. 2021 Oct 1;12:718503. doi: 10.3389/fgene.2021.718503 (PMC8517332; doi:10.3389/fgene.2021.718503)
Supplement: Supplementary file 1 [file DataSheet1.doc]

**Supplemental material**


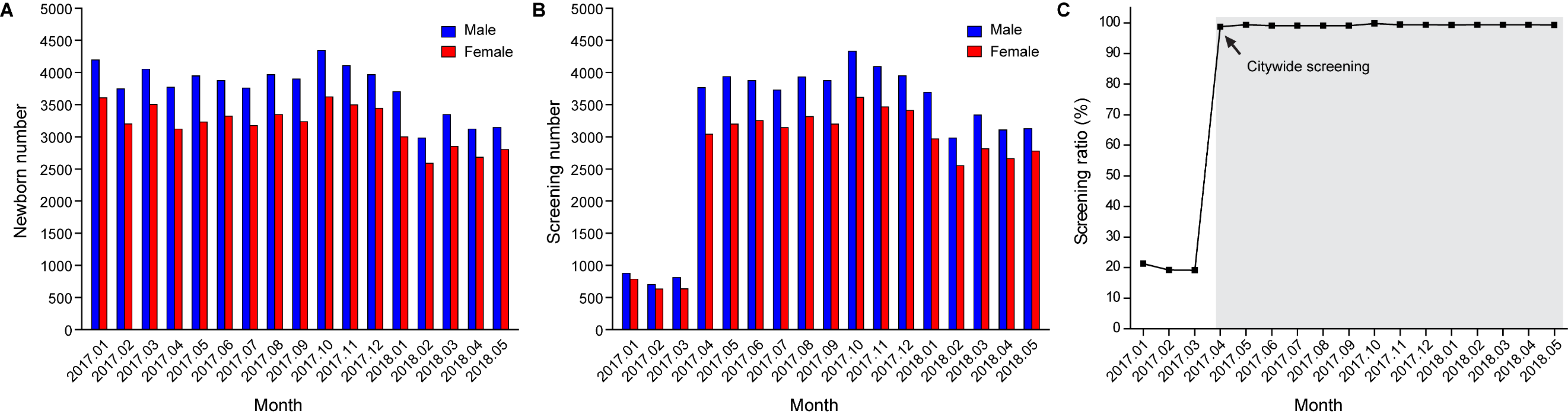


**Supplemental Figure 1.** Newborn number (A), screening number (B), and screening ratio (C) in Xiamen from January 2017 to May 2018.


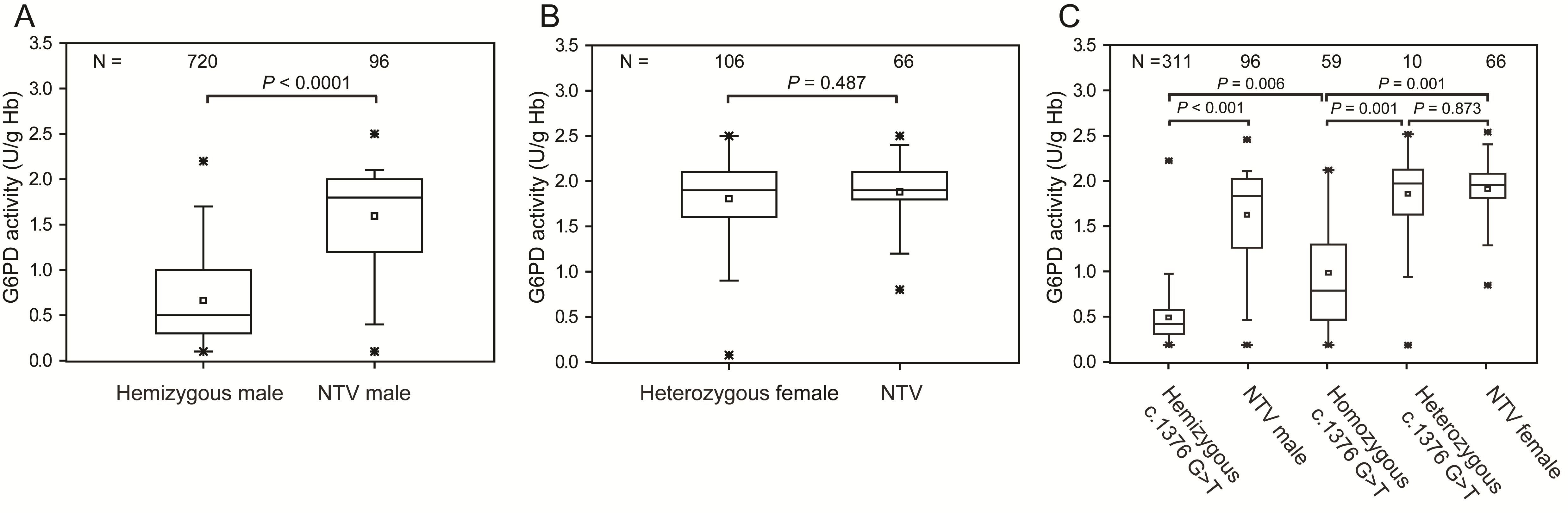


**Supplemental Figure 2.** Statistical analysis of G6PD activities for hemizygous male (A), heterozygous female (B), and newborns carrying c.1376 G>A variant (C). The line within the box denotes the median, the square within the box denotes the mean, the horizontal borders of each box denote the 25th and 75th percentiles, the whiskers denote the 5th and 95th percentiles, and the stars denote the maximum and minimum. NTV, negative for target variants.

**Supplemental Table 1.** PCR Cycling conditions in G6PD genotyping test.

|  | **Conditions** | **Cycle number** |
| --- | --- | --- |
| PCR amplification | 50 °C, 2min | 1 |
|  | 95 °C, 10min | 1 |
|  | 95 °C, 15s | 10 |
|  | 65 °C, 15s (-1 °C/cycle), data collection. |  |
|  | 76 °C, 20 s |  |
|  | 95 °C, 15 s | 50 |
|  | 55 °C, 15 s |  |
|  | 76 °C, 20 s |  |
| Melting curve analysis | 95 °C, 1 min | 1 |
|  | 37 °C, 3 min |  |
|  | 40 °C ~ 85 °C (at a ramp rate of 0.5 °C/step), data collection. |  |

**Supplemental Table 2.** Distribution of different G6PD genotypes and the corresponding G6PD activities.

| **Genotype** | **Male**  **hemizygote** | **G6PD activity**  **(U/g Hb) ^†^** | **Female**  **heterozygote** | **G6PD activity**  **(U/g Hb) ^†^** | **Female**  **homozygote** | **G6PD activity**  **(U/g Hb) ^†^** |
| --- | --- | --- | --- | --- | --- | --- |
| c.95A>G | 68 | 0.6 ± 0.4, 0.1-1.4 | 10 | 1.8 ± 0.6, 0.1-2.1 | 0 | - |
| c.383T>C | 1 | 0.5 | 1 | 1.2 | 0 | - |
| c.392G>T | 37 | 1.4 ± 0.4, 0.4-2.0 | 2 | 1.4 ± 0.3, 1.2-1.7 | 1 | 1.2 |
| c.487G>A | 18 | 0.8 ± 0.3, 0.2-1.4 | 2 | 1.7 ± 0.1, 1.6-1.9 | 0 | - |
| c.517T>C | 2 | 1.4 ± 0.1, 1.3-1.5 | 1 | 2.5 | 0 | - |
| c.592C>T | 1 | 0.1 | 0 | - | 0 | - |
| c.871G>A | 35 | 0.7 ± 0.3, 0.1-1.3 | 2 | 1.3 ± 1.0, 0.3-2.4 | 2 | 1.9 ± 0.0, 1.9-1.9 |
| c.1004C>A | 1 | 1.2 | 0 | - | 0 | - |
| c.1024C>T | 59 | 1.4 ± 0.4, 0.1-2.1 | 3 | 1.7 ± 0.2, 1.6-2.0 | 2 | 1.8 ± 0.7, 1.1-2.5 |
| c.1360C>T | 17 | 0.2 ± 0.5, 0.1-2.1 | 6 | 1.7 ± 0.1, 1.6-1.9 | 1 | 0.1 |
| c.1376G>T | 311 | 0.4 ± 0.3, 0.1-2.2 | 59 | 1.8 ± 0.5, 0.1-2.5 | 10 | 0.9 ± 0.7, 0.1-2.1 |
| c.1388G>A | 170 | 0.8 ± 0.4, 0.1-1.9 | 20 | 1.8 ± 0.4, 0.3-2.5 | 3 | 1.1 ± 0.8, 0.2-2.1 |
| c.1376G>T/c.1388G>A | 0 | - | 2 | 0.6 ± 0.3, 0.3-1.0 | 0 | - |
| c.871G>A/c.1376G>T | 0 | - | 1 | 1.6 | 0 | - |
| c.871G>A/c.1388G>A | 0 | - | 2 | 1.2 ± 0.2, 0.9-1.4 | 0 | - |
| c.95A>G/c.1376G>T | 0 | - | 1 | 0.7 | 0 | - |
| Mutation unknown^‡^ | 96 | 1.6 ± 0.6, 0.1-2.5 | 66 | 1.9 ± 0.3, 0.8-2.5 |  |  |
| Total | 816 |  | 178 |  | 19 |  |

**^†^**The G6PD activity (U/g Hb) was presented as mean ± SD and range.

^‡^Mutation unknown indicates that subjects are negative for target variants in MeltPro® G6PD genotyping test.

**Supplemental Table 3.** Classification and distribution of G6PD activities in 851 neonates based on the WHO guideline.

| **Genotype** | **WHO classification for males with mutation** | | | **WHO classification for females with mutation** | | |
| --- | --- | --- | --- | --- | --- | --- |
|  | **Ⅱ**  **(≤ 0.5 U/g Hb)** | **Ⅲ**  **(0.5-2.8 U/g Hb)** | **Ⅳ**  **(2.8-7.1 U/g Hb)** | **Ⅱ**  **(≤ 0.5 U/g Hb)** | **Ⅲ**  **(0.5-2.8 U/g Hb)** | **Ⅳ**  **(2.8-7.1 U/g Hb)** |
| c.95 A>G | 36 (52.9, 0.3) | 32 (47.1, 0.9) | 0 | 1 (10.0, 0.1) | 9 (90.0, 2.0) | 0 |
| c.383 T>C | 1 (100, 0.5) | 0 | 0 | 0 | 1 (100, 1.2) | 0 |
| c.392 G>T | 1 (2.7, 0.4) | 36 (97.3, 1.5) | 0 | 0 | 2 (100, 1.4) | 0 |
| c.487 G>A | 4 (22.2, 0.3) | 14 (77.8, 1.0) | 0 | 0 | 2 (100, 1.7) | 0 |
| c.517 T>C | 0 | 2 (100, 1.4) | 0 | 0 | 1 (100, 2.5) | 0 |
| c.592 C>T | 1 (100, 0.1) | 0 | 0 | 0 | 0 | 0 |
| c.871 G>A | 12 (34.3, 0.4) | 23 (65.7, 0.8) | 0 | 1 (50.0, 0.3) | 1 (50.0, 2.4) | 0 |
| c.1004 C>A | 0 | 1 (100, 1.2) | 0 | 0 | 0 | 0 |
| c.1024 C>T | 2 (3.4, 0.2) | 57 (96.6, 1.4) | 0 | 0 | 3 (100, 1.7) | 0 |
| c.1360 C>T | 16 (94.1, 0.1) | 1 (5.9, 2.1) | 0 | 0 | 6 (100, 1.7) | 0 |
| c.1376 G>T | 248 (79.7, 0.3) | 63 (20.3, 0.8) | 0 | 2 (3.4, 0.2) | 57 (96.6, 1.9) | 0 |
| c.1388 G>A | 48 (28.2, 0.3) | 122 (71.8, 0.9) | 0 | 1 (5.0, 0.3) | 19 (95.0, 1.9) | 0 |
| Compound mutations | 0 | 0 | 0 | 1 (16.7, 0.3) | 5 (83.3, 1.1) | 0 |
| Homozygous mutations | 0 | 0 | 0 | 5 (26.3, 0.2) | 14 (73.7, 1.4) | 0 |

Data were shown as number (percentage, mean value of enzyme activity). Median value (4.7 U/g Hb) obtained from normal male subjects in our study considered as normal G6PD activity. According to WHO standard, the residual enzyme activity ≤ 0.5 U/g Hb (10% of normal) was grouped into class II (severe deficiency); the residual enzyme activity between 0.5-2.8 U/g Hb (10-60% of normal) was grouped into class III (moderate deficiency), the residual enzyme activity between 2.8-7.1 U/g Hb (60-150% of normal) was grouped into class IV (normal).
